# Comparative Effectiveness of Single‐Dose vs. Multi‐Dose Prophylactic Antibiotics in Reducing Post‐Surgical Infections: A Systematic Review and Meta‐Analysis

**DOI:** 10.1155/ijm/8368761

**Published:** 2026-05-21

**Authors:** Sariya Khan, Mariam Amro, Muddassir Khalidi, Sadia Sultana, Mable Pereira, Manal El Said

**Affiliations:** ^1^ General Medicine Practice Program, Batterjee Medical College, Jeddah, Saudi Arabia, bmc.edu.sa; ^2^ Bsc in Software Engineering, College of Computer Science and Information Systems, Prince Sultan University, Riyadh, Saudi Arabia, psu.edu.sa; ^3^ School of Medicine, Lincoln American University, Georgetown, Demerara-Mahaica, Guyana; ^4^ Department of Microbiology and Infection Prevention & Control Unit, Theodor Bilharz Research Institute, Giza, Egypt, tbri.sci.eg

**Keywords:** multiple dose, perioperative prophylaxis, prophylaxis, single dose, surgical site infections

## Abstract

**Background:**

Surgical site infections (SSIs) are among the most frequent and costly postoperative complications, contributing to increased morbidity, extended hospitalization, and healthcare burden. Although guidelines support single‐dose antibiotic prophylaxis for most surgeries, multi‐dose regimens remain widely used in clinical practice, especially in high‐risk procedures. This review aimed to compare the effectiveness of single‐dose versus multiple‐dose prophylactic antibiotic regimens in preventing postoperative infections.

**Methods:**

A systematic review and meta‐analysis were conducted according to PRISMA 2020 guidelines. Databases including PubMed, Scopus, CENTRAL, Web of Science, and ScienceDirect were searched up to February 2025. Studies involving human participants undergoing surgery, comparing single‐ and multiple‐dose antibiotic prophylaxis with infection‐related outcomes, were included. Risk of bias was assessed using the Cochrane RoB 2.0 and MINORS tools. Meta‐analyses were performed using random‐effects models.

**Results:**

Forty‐seven studies encompassing various surgical disciplines and patient populations were included. No statistically significant difference in SSI rates was observed between single‐ and multiple‐dose groups (OR: 1.10 [95% CI: 0.87–1.39], *p* = 0.43). Similarly, no differences were detected for other postoperative infections, mortality, or readmissions. Heterogeneity was moderate to high across analyses. Most studies reported comparable infection outcomes despite differences in antibiotic type, follow‐up duration, and surgical setting.

**Conclusion:**

Single‐dose antibiotic prophylaxis is as effective as multiple‐dose regimens in reducing postoperative infections in most surgical contexts. These findings support current recommendations favoring single‐dose administration and reinforce antimicrobial stewardship efforts. Extended prophylaxis should be reserved for selected high‐risk cases rather than routine use.

## 1. Introduction

Surgical site infections (SSIs) stand as one of the most common surgical complications that trigger elevated morbidity rates, prolonged hospitalization, and elevated healthcare costs worldwide. Before surgery, prophylactic antibiotics are frequently given to avoid SSIs. However, there is an ongoing discussion on the best dosage approach, including whether to use a single preoperative dose or a multi‐dose regimen that lasts into the postoperative phase. Leading health organizations have established official guidelines about this practice. The World Health Organization (WHO) suggests giving prophylactic antibiotics 120 min before skin incision while considering antibiotic half‐life to reach effective tissue concentrations during surgery [1]. The Centers for Disease Control and Prevention (CDC) recommends prophylactic antibiotics administration when bactericidal concentrations reach the tissue at the time of incision [2]. The recommended time of antibiotic administration is 1 h before to incision unless vancomycin or fluoroquinolones are being used, in which case the window is extended to 2 h. For the majority of surgical operations, the CDC advises a single preoperative antibiotic dosage; however, if the surgery takes longer than expected or there is significant bleeding, extra intraoperative doses are required [2].

The recommendations support single‐dose prophylaxis for most surgeries but multiple‐dose regimens persist across many procedures especially when implants are used or infection risks are high [3]. The current practice deviates from the recommendations found in guidelines thus creating a mismatch between evidence‐based recommendations and real‐world clinical procedures.

Prior research studies show conflicting results because some reports indicate both approaches yield similar outcomes while others recommend extended dosing under particular clinical situations [3, 4]. A thorough investigation of available evidence becomes necessary due to ongoing medical discussions about antibiotic dosing protocols. The analysis evaluates the infection prevention outcomes between single‐dose and multiple‐dose prophylactic antibiotic administration methods. The analyzed studies reached 47 in number while including diverse surgical specialties and patient demographics. The review uses existing data to provide clinical evidence for decision support and antimicrobial stewardship optimization in surgical practice.

With the global emphasis on antimicrobial stewardship and the need to decrease SSIs, as well as antibiotic utilization, finding the most appropriate manner to perform prophylactic dosing is imperative for evidence‐based surgery. Even though single‐dose protocols have advantages regarding cost, compliance, and reduced resistance, the multi‐dose protocols remain widespread use, particularly in contaminated or high‐risk procedures. Current guidelines remain neither consistent nor harmonious, and no clear consensus regarding superiority exists.

Thus, the current systematic review has aimed to contrast in detail the effectiveness of single‐dose versus multi‐dose prophylactic antibiotic regimens in reducing post‐surgical infections across broad areas of surgery and patient populations. Through meta‐analysis of available clinical data, this review has hoped to allow more consistent, rational, and cost‐efficient use of antibiotics during the perioperative period.

## 2. Methodology

### 2.1. Protocol Approval and Registration

This systematic review and meta‐analysis was designed in accordance with the Preferred Reporting Items for Systematic Reviews and Meta‐Analyses (PRISMA) 2020 guidelines [5]. The protocol for this study was registered in the International Prospective Register of Systematic Reviews (PROSPERO) under the registration number CRD420250653720. As this investigation involved secondary analysis of previously published data, ethical approval and informed consent were not necessary.

### 2.2. Search Strategy

A comprehensive literature search was conducted across PubMed, Scopus, and ScienceDirect from database inception to February 15, 2025. The search strategy was constructed using a combination of MeSH terms and free‐text keywords including: “prophylactic antibiotics,” “antibiotic prophylaxis,” “single‐dose,” “multiple‐dose,” “repeated doses,” “surgical site infection,” “SSI,” and “postoperative infection.” Boolean operators were applied to optimize and expand the search yield. An example search strategy included the following: (“Prophylactic Antibiotics” OR “prophylactic antibiotic” OR “antibiotic prophylaxis”) AND (“single‐dose” OR “single dose” OR “single administration” OR “single‐shot antibiotic”) AND (“multi‐dose” OR “multiple‐dose” OR “multiple doses” OR “repeated doses”) AND (“post‐surgical infection” OR “surgical site infection” OR “SSI” OR “postoperative infection”). In total, 838 records were retrieved. After duplicate removal, 773 unique records were included for screening. Additionally, the reference lists of all eligible studies and relevant systematic reviews were manually scanned to identify any studies that may have been missed by the database searches.

### 2.3. Study Selection Criteria

Studies were considered eligible if they were published in English, involved human participants of any age, and reported undergoing any surgery. Only original research articles including randomized controlled trials (RCTs), prospective or retrospective cohort studies, and cross‐sectional studies were included. Studies were excluded if they were case reports, case series, narrative or systematic reviews, editorials, in vitro experiments, animal studies, or if they did not clearly define infection‐related outcomes.

### 2.4. Screening

All records retrieved from the database search were imported into Rayyan, a web‐based software for systematic review management [6]. Two reviewers independently screened titles and abstracts to identify potentially eligible studies. Full‐text screening of selected articles was then performed independently by the same reviewers. Any disagreements regarding inclusion were resolved through discussion, and a third reviewer was consulted when necessary to reach consensus.

### 2.5. Data Extraction and Risk of Bias Assessment

Data extraction was performed independently by two researchers using a pre‐designed standardized form. Extracted information included the following: study title, author names, year of publication, country, journal, study design, sample size, type of surgery, participant demographics, type and dose of antibiotics used, duration of administration (single vs. multiple), and infection‐related outcomes. The risk of bias was assessed independently using appropriate tools based on study design. The Cochrane Risk of Bias 2.0 tool was applied for RCTs, while the Methodological Index for Non‐Randomized Studies (MINORS) were used for cohort and cross‐sectional studies [7, 8]. Studies deemed to be at high or critical risk of bias were excluded from the final meta‐analysis to ensure methodological rigor. Formal assessment of publication bias using funnel plots or statistical tests (e.g., Egger’s test) was not performed. Given the substantial clinical and methodological heterogeneity across included studies, as well as variability in outcome definitions and study designs, interpretation of funnel plot asymmetry would be unreliable.

### 2.6. Outcome Measures

The primary outcome of interest was the incidence or rate of post‐surgical infections, including SSI, wound infections, or other documented postoperative infectious complications. Secondary outcomes included the timing of infections, severity, need for reoperation or antibiotic escalation, and hospital readmission due to infection. Subgroup analysis was planned based on the type of surgery (e.g., orthopedic, abdominal, gynecologic) and antibiotic class. Where reported, the duration of multiple‐dose prophylactic regimens was extracted. However, reporting of regimen duration was highly inconsistent across studies, ranging from limited postoperative dosing to multi‐day courses, and was frequently insufficiently detailed to allow standardized categorization.

### 2.7. Data Analysis

Effect estimates, including odds ratios (ORs) with 95% confidence intervals (CIs), were extracted directly or calculated from raw data. Meta‐analyses were conducted using the random‐effects model to account for anticipated heterogeneity among studies. Statistical heterogeneity was quantified using the I^2^ statistic, with thresholds of 25%, 50%, and 75% indicating low, moderate, and high heterogeneity, respectively. All statistical analyses were performed using Review Manager (RevMan) version 5.4.

## 3. Results

**Figure 1:**
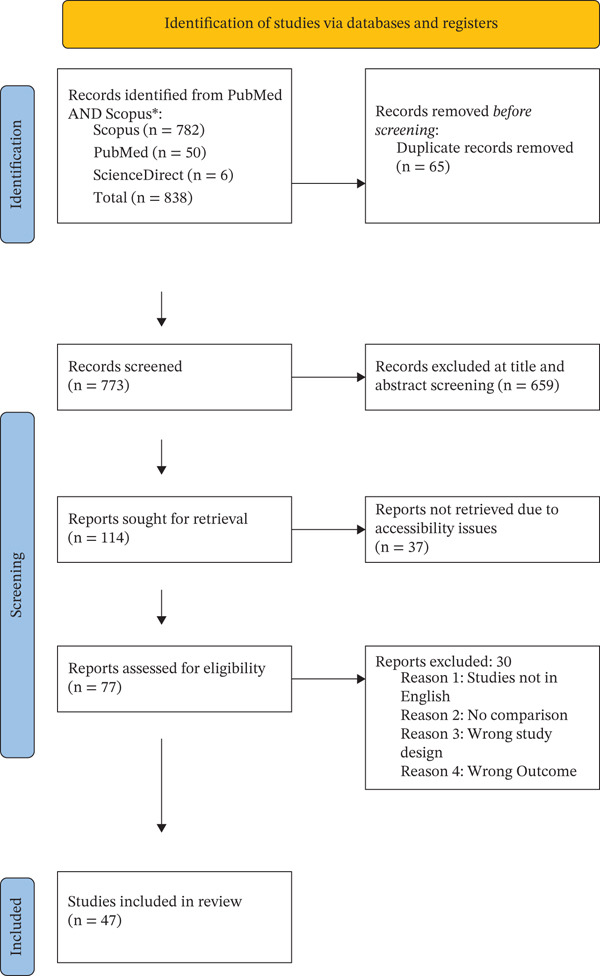
PRISMA Flowchart.

A total of 838 records were identified through database searches. After removing 65 duplicates, 773 records were screened by title and abstract. Of these, 659 records were excluded due to irrelevance to the research question. The remaining 114 full‐text articles were assessed for eligibility, out of which, 37 reports were not retrieved due to accessibility issues. Of the 77 full‐text articles assessed for eligibility, 30 were excluded for the following reasons: not published in English (n = 8), absence of a comparator group (n = 6), inappropriate study design (n = 4), or lack of relevant or extractable outcome data (n = 12). Finally, 47 studies met the inclusion criteria and were included in the systematic review as shown in Figure 1.

A total of 47 studies were included in the qualitative synthesis as shown in Table 1, encompassing a wide range of surgical specialties and methodological designs. The included studies originated from geographically diverse regions, including high‐income countries such as the United States, United Kingdom, Canada, Japan, and Australia, as well as several from low‐ and middle‐income countries including Pakistan, Iran, Iraq, Tanzania, and India. Study designs varied, with the majority being RCTs (*n* = 23), complemented by retrospective cohort studies (*n* = 13), prospective cohorts (*n* = 7), and quasi‐experimental and observational designs. Sample sizes varied markedly, ranging from as few as 20 participants [46] to over 15,000 patients [27] reflecting heterogeneity in study scale.

**Table 1:** General characteristics of included studies.

Study ID (author, year)	Country	Study design	Total number of participants		Gender distribution (Female%)	Mean age	Type of surgery	Antibiotic used
			SD	MD				
Abro,2014 [9]	Pakistan	Randomized prospective study	419	419	38	68.2 ± 10.5	CABG and/or valve surgery	Cefazolin
Akkour, 2020 [10]	Saudi Arabia	Retrospective cohort study	117	116	56.2	—	Instrumented lumbar spinal fusion	Cefazolin
Andy, 2014 [11]	USA	Retrospective cohort study	80	80	58.8	50.59	Elective laparoscopic cholecystectomy	Cefazolin
Badge, 2022 [12]	Australia	Prospective multicenter cohort study	292	534	55.6	47.8	ORIF for closed ankle fractures	First‐generation Cephalosporin (90% of patients), Clindamycin (9%), Vancomycin (1%)
Bashir, 2024 [13]	Pakistan	Retrospective cohort study	1222	650	41.07	64.5	MIS for colorectal cancer	Cefotetan (IV)
Bates, 1992 [14]	UK	RCT	345	353	100	46.5	Implant‐based breast reconstruction	Cloxacillin (2 g per dose); Clindamycin (600 mg per dose) for penicillin‐allergic patients
Chang, 2008 [15]	Taiwan	Retrospective cohort study	530	102	57.3	35	Kidney transplant donor surgery	First‐generation cephalosporins (91.8%), Fourth‐generation cephalosporins (8.2%)
Christensen, 2021 [16]	USA	Retrospective case–control study	399	399	64.3	48.78 ± 10.71	Laparoscopic cholecystectomy	Ceftriaxone (1 g IV)
Fryberger, 2021 [17]	USA	Retrospective cohort study	68	73	100	55.14 ± 9.43	Elective hysterectomy	Cefazolin (2–3 g IV)
Fujita, 2007 [18]	Japan	RCT	126	896	—	67.57 ± 9.88	TSA, rTSA	Cefazolin (first‐line) or Vancomycin (for allergies)
Gahm, 2022 [19]	Sweden	RCT	150	150	44.3	10 months	Pediatric neurosurgical interventions	Cephalothin (first‐generation cephalosporin)
Galask, 1988 [20]	USA	RCT	187	187	76.74	37.74 ± 6.40	Laparoscopic cholecystectomy	3rd generation Cephalosporin (Ceftriaxone)
Haga, 2012 [21]	Japan	Prospective randomized study	554	2763	54.05	66.11 ± 10.25	THA, TKA	Cefazolin, Vancomycin, Clindamycin
Haider, 2022 [22]	Pakistan	Comparative prospective study	—	—	—	67.6	THA, TKA	Cephalosporins: Cefazolin, Cefotaxime
Han, 2014 [23]	South Korea	Retrospective cohort study	51	60	36.9	24.5 ± 2.2	Open appendectomy	Metronidazole (500 mg IV)
Haverkorn, 1987 [24]	Netherlands	Double‐Blind, placebo‐controlled clinical trial	76	76	—	28.30 ± 10.70	Open appendectomy	Ceftriaxone (1 g IV) + Metronidazole (500 mg IV)
Hellbusch, 2008 [25]	USA	Prospective randomized clinical study	40	256	49.6	64	UKA	First‐generation cephalosporin (Cefazolin preferred); alternative: vancomycin or clindamycin
Hussain, 2012 [26]	Saudi Arabia	RCT	595	1327	44.01	—	ISS and NSS	Cefazolin alternative: Ciprofloxacin (for allergies)
Ishibashi, 2014 [27]	Japan	Prospective randomized non‐inferiority trial	6540	8864	—	—	Trauma surgery	Flucloxacillin + gentamicin, cefuroxime
Jabeen, 2007 [28]	Pakistan	RCT	201	1129	32.6	61.3 ± 12.1	Radical gastrectomy for gastric carcinoma	Cefazolin
Jagelman, 1988 [29]	USA	RCT	192	198	51.5	22.6	Open appendectomy	Cefuroxime sodium + Metronidazole
Kanellakopoulou, 2009 [30]	Greece	Prospective, open‐label, randomized study	250	250	100	—	Emergency C‐section	Gentamicin + Metronidazole
Kasatpibal, 2006 [31]	Thailand	Prospective cohort study	104	104	53.4	34.7	GS	Ceftriaxone (2 g IV)
Ku, 2024 [32]	South Korea	Retrospective study with PSM	139	140	35.4	65	Elective rectal cancer surgery	Flomoxef
Lyimo, 2013 [33]	Tanzania	RCT	154	306	100	60.3 ± 10.1	Pelvic organ prolapse surgery with graft/mesh	Cefazolin (77%) Cefazolin + Gentamicin/Metronidazole (5%) Alternative Antibiotics for Allergy: Gentamicin/Clindamycin (11%), Vancomycin (3%), Levofloxacin (1%), Doxycycline (< 1%)
Lyimo, 2012 [34]	Tanzania	RCT	89	87	100	26.2 ± 7.0	C‐section	Ampicillin + Metronidazole
Maciejczak, 2019 [35]	Poland	Prospective non‐randomized cohort study	256	312	83.2	70.69 ± 8.01	THA and TKA	Teicoplanin (single‐dose) second‐generation cephalosporins, *β*‐lactam/*β*‐lactamase inhibitors, or ciprofloxacin (multi‐dose)
Mangan, 2025 [36]	USA	Retrospective cohort study	179	181	46.6	66 ± 9	Elective colon cancer surgery	Flomoxef
Mehta, 1990 [37]	India	Prospective observational study	164	161	27.6	68	Elective gastric cancer surgery	Cefazolin
Mohammadi, 2021 [38]	Iran	Nonrandomized, historically controlled, equivalence trial	147	163	100	41.2 ± 5.8	LAVH	Cefazolin
Mohri, 2007 [39]	Japan	RCT	481	481	—	—	C‐section	Gentamicin + Metronidazole
Rafiq, 2015 [40]	Pakistan	RCT	195	182	45.8	31.70 ± 9.96	Open appendectomy	Cefuroxime Sodium + Metronidazole
Roex, 1987 [41]	Netherlands	RCT	48	47	65.2	26	Third molar surgery	Amoxicillin
Sadraei‐Moosa, 2017 [42]	Iran	RCT	190	187	38.1	62.1 ± 9.8	Elective colorectal surgery	Cefmetazole
Saginur, 2000 [43]	Canada	RCT	766	813	53.1	26	Open appendectomy	Metronidazole and gentamicin
Salih, 2018 [44]	Iraq	Prospective comparative study	243	243	24.1	68	Elective gastric cancer surgery	Cefazolin or Ampicillin–sulbactam
Schmidt‐Matthiesen, 1999 [45]	Germany	Prospective, RCT (Phase IV)	69	69	—	39	Abdominoplasty	Cephalosporin (1 g IV) or Levofloxacin (500 mg IV) if allergic
Sevin, 2007 [46]	Turkey	Prospective cohort	20	20	100	—	Elective abdominal hysterectomy	Cephradine
Shah, 1998 [47]	UAE	RCT	46	47	100	—	C‐section	Piperacillin, Cephradine + Metronidazole
Siddiqi, 2010 [48]	New Zealand	RCT	449	451	—	54.4	Abdominal surgery	Augmentin (Amoxicillin 250 mg + Clavulanic Acid 125 mg)
Suzuki, 2011 [49]	Japan	RCT	1518	1509	20.05	61.4 ± 10.2	CABG, valve replacement/repair, or both	Teicoplanin (Single‐Dose), Cefazolin (Multi‐Dose)
Tamayo, 2008 [50]	Spain	Randomized prospective clinical study	97	98	19.4	37	Emergency surgery for penetrating trauma	Ceftriaxone (Single‐Dose), Cefoxitin (Multi‐Dose)
Thareja, 2024 [51]	India	Single‐center prospective observational cohort study	78	235	100	47	Hysterectomy (vaginal or abdominal), with or without colporrhaphy	Metronidazole
Warnock, 2019 [52]	Northern Ireland	Prospective observational cohort study	66	72	100	29 ± 4.1	C‐section	Cefoxitin
Westen, 2015 [53]	Tanzania	RCT	164	75	44.3	—	Elective colorectal surgery	Cefotetan (single‐dose) vs. Cefoxitin (multi‐dose)
Wyles, 2019 [54]	USA	Retrospective cohort study	162	79	100	—	C‐section	Cefotetan (single‐dose) vs. Cefoxitin (multi‐dose)
Zafar, 2024 [55]	Pakistan	Quasi‐experimental study	195	253	56.6	26.2	Cardiac surgery (closed and open‐heart procedures)	Ampicillin + Gentamicin vs. Cefazolin + Gentamicin

Surgical procedures represented in the dataset spanned across multiple domains, including orthopedic surgeries (e.g., total knee or hip arthroplasty), gastrointestinal and colorectal procedures, cardiac surgery (e.g., CABG, valve repair), obstetric and gynecologic operations (e.g., C‐sections, hysterectomies), neurosurgery, plastic surgery, and emergency trauma interventions. The age of participants ranged from pediatric patients as young as 10 months [19] to elderly individuals undergoing colorectal or cardiac surgeries, with mean age values commonly falling between the 40s and 60s. Gender distribution also varied, with several studies focused exclusively on female patients due to surgical context (e.g., hysterectomy or cesarean delivery).

Antibiotics used across the included studies showed notable variability. First‐generation cephalosporins such as cefazolin were the most commonly administered prophylactic agents, but a variety of regimens were employed depending on institutional protocols and surgical settings. These included combinations such as cefuroxime with metronidazole, flucloxacillin with gentamicin, as well.

### 3.1. Risk of Bias Assessment

The Cochrane Risk of Bias 2.0 (RoB 2) tool was used to evaluate the methodological quality of RCTs included in this review (Table S1 attached in the Supporting Information). The majority of RCTs were judged to be at low risk of bias across all five domains. Notably, 19 out of 23 studies demonstrated low risk in domains such as bias arising from the randomization process, deviations from intended interventions, and outcome measurement. However, a few studies presented methodological limitations: one study [40] was rated as having a high risk of bias due to deviations from intended interventions and outcome measurement, while two others [42, 50] raised some concerns in multiple domains. Overall, the randomized studies were deemed to be of acceptable quality, with most considered suitable for inclusion in meta‐analyses.

The Methodological Index for Non‐Randomized Studies (MINORS) tool was applied to assess the risk of bias in non‐randomized comparative and non‐comparative studies (Tables S2 and S3 attached in the Supporting Information). Among non‐comparative studies (*n* = 2), both scored 11 out of a possible 16 points, with minor concerns noted in the domains of follow‐up duration, endpoint assessment, and prospective calculation of study size. For the 20 non‐randomized comparative studies assessed, scores ranged from 12 to 20 (maximum 24), indicating moderate to high methodological quality overall. Most studies clearly stated their aims, used appropriate endpoints, and had acceptable comparability between groups. However, some studies lacked prospective data collection, adequate follow‐up information, and clarity regarding sample size justification. Despite these limitations, the methodological rigor of the non‐randomized studies was generally robust, allowing their inclusion in the qualitative synthesis.

**Figure 2:**
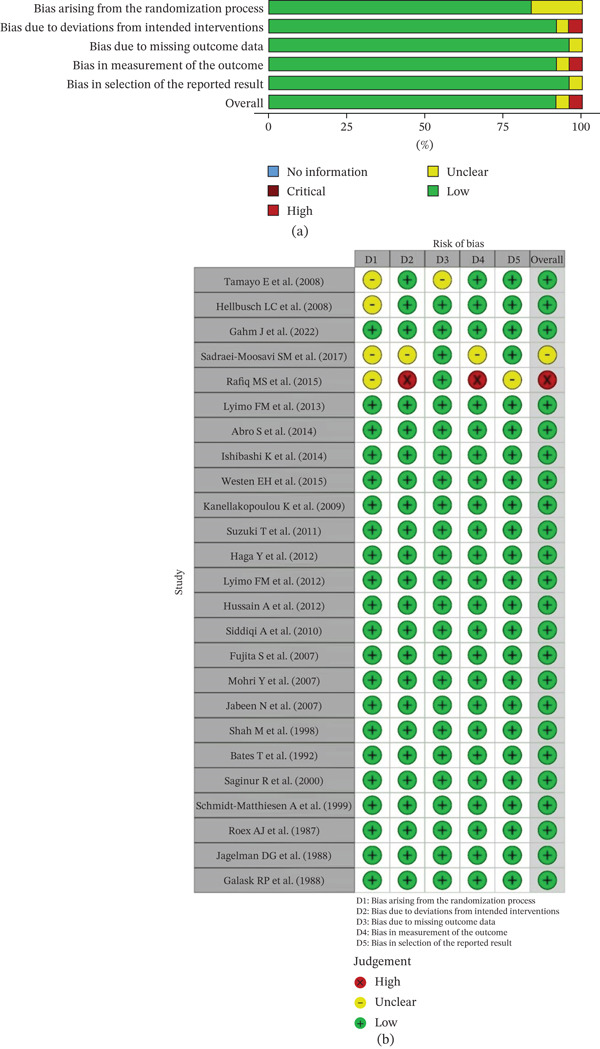
RoB assessment using the Cochrane RoB 2 tool for randomized controlled trials. (a) Summary plot of domain‐level judgments across studies. (b) Traffic light plot showing individual study‐level assessments.

**Figure 3:**
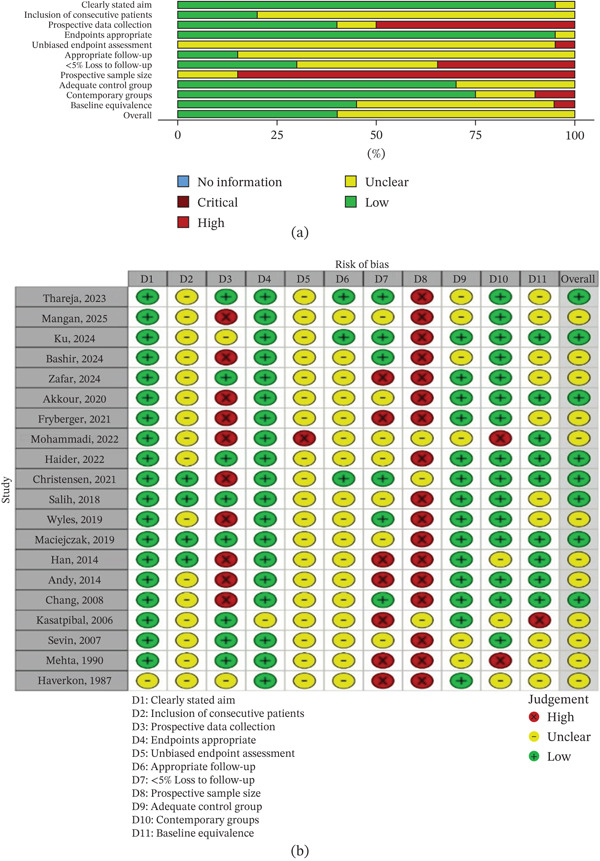
RoB assessment using the MINORS tool for non‐randomized comparative studies. (a) Summary plot of domain‐level scores across studies. (b) Traffic light plot showing individual study‐level assessments.

**Figure 4:**
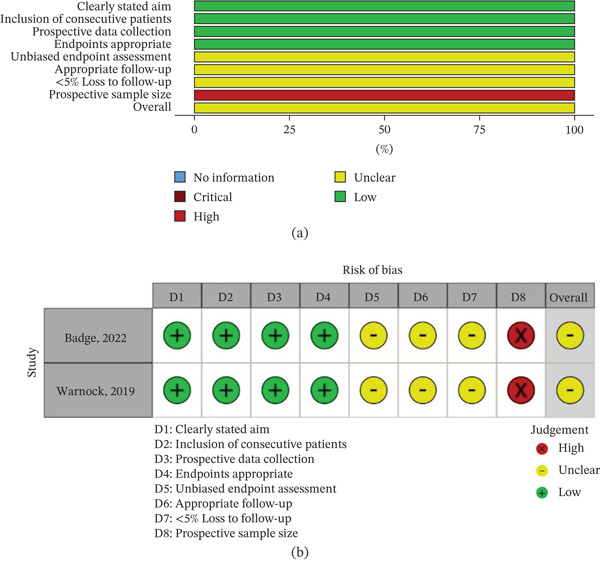
RoB assessment using the MINORS tool for non‐randomized non‐comparative studies. (a) Summary plot of domain‐level scores across studies. (b) Traffic light plot showing individual study‐level assessments.

Figures 2a,b, 3a,b and 4a,b summarize the RoB assessments for both RCTs and non‐randomized studies.

### 3.2. Primary Outcomes

**Figure 5:**
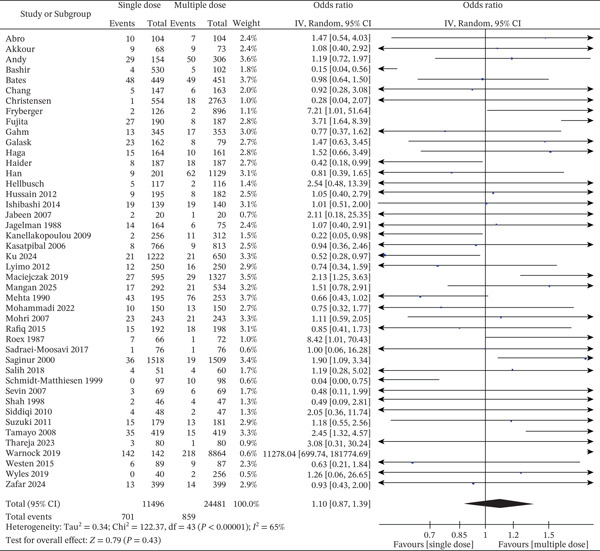
Forest plot of surgical site infection incidence comparing single‐dose versus multiple‐dose prophylactic antibiotics (44 studies; random‐effects model). The pooled effect estimate is presented as ORs with 95% CIs. Statistical heterogeneity was assessed using the I^2^ statistic. The *x*‐axis represents ORs on a logarithmic scale.

Figure 5 displays the pooled results of 44 studies depicting the difference between SSI rate in patients receiving single‐dose vs. multiple‐dose antibiotic prophylaxis treatment regimens. In all the incorporated studies together, 11,496 patients received single‐dose antibiotics and 24,481 multiple‐dose regimens. The pooled OR was 1.10 [95% CI: 0.87–1.39], and no statistically significant difference between the two dosing regimens existed (*Z* = 0.79, *p* = 0.43). There was important heterogeneity across studies (*I* ^2^ = 65*%*, *τ* ^2^ = 0.34, *p* < 0.00001), meaning important difference in study design, population, surgery done, or outcome definitions for infection. Note is the important difference in event rates between 0% and over 30% in both groups, with a few studies [17, 41] having very wide CIs due to low numbers or uncommon events. While a number of studies [14, 45] indicated a tendency towards a single‐dose regime, others [9, 22, 36] showed better results with more doses. None of those specific findings markedly altered the overall effect estimate. The diamond marker of the plot crosses the no‐effect line, which visually verifies the lack of statistical significance. These findings suggest that, across a range of different surgical environments, single‐dose and multiple‐dose prophylaxis regimens yield equivalent SSI rates in favor of current antimicrobial stewardship programs aimed at minimizing unnecessary antibiotic exposure without compromise of infection control. Although 47 studies met inclusion criteria for qualitative synthesis, only 44 studies were eligible for the primary meta‐analysis of SSI outcomes. Three studies were excluded from this pooled estimate due to non‐comparative design, absence of extractable event data, or reporting outcomes in a format unsuitable for meta‐analysis. Eligibility for each pooled analysis varied according to outcome‐specific data availability.

**Figure 6:**
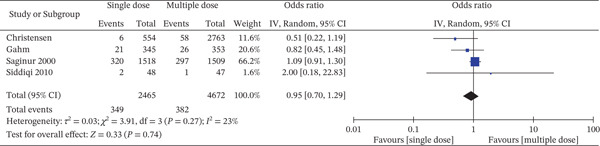
Forest plot of other post op infection incidence comparing single‐dose versus multiple‐dose prophylactic antibiotics (4 studies; random‐effects model). The pooled effect estimate is presented as ORs with 95% CIs. Statistical heterogeneity was assessed using the I^2^ statistic. The *x*‐axis represents ORs on a logarithmic scale.

Four studies compared the incidence of other post‐operative infections in patients receiving single‐dose and multiple‐dose antibiotic prophylaxis regimens (Figure 6). The pooled analysis consisted of 349 events among 2465 patients in the single‐dose group and 382 events among 4672 patients in the multiple‐dose group. The pooled OR was 0.95 [95% CI: 0.70–1.29], which represents no statistically significant difference in the risk of other post‐operative infections between the two regimens of antibiotics (*Z* = 0.33, *p* = 0.74). This finding illustrates clinical equipoise for infection outcomes when single‐dose is compared with multiple‐dose prophylaxis. Of interest, the CI crossed the line of no effect (*OR* = 1), and the overall effect test was not significant.

Study‐level effect estimates varied. Christensen et al. found an OR of 0.51 [0.22–1.19] in favor of single‐dose prophylaxis, while Siddiqi et al. found a much larger OR of 2.00 [0.18–22.63], suggesting a potential excess risk with single‐dose, though with a very broad CI for imprecision due to small sample size [16, 48]. Gahm et al. (*OR* = 0.82 [0.45–1.48]) also favored single‐dose, while Saginur et al. (*OR* = 1.09 [0.91–1.30]) indicated a slight but non‐significant preference for the multiple‐dose strategy [19, 43].

Weighting in the meta‐analysis was dominated by Saginur et al. (66.2%), the biggest and most precise study in the analysis, which would have tended to influence the neutrality of the pooled estimate [43]. On the other hand, smaller studies such as Siddiqi et al. (1.6% weight) had very minimal contribution due to lack of power [48]. Statistical heterogeneity was low to moderate (*I* ^2^ = 23*%*, *τ* ^2^ = 0.03, *C* *h* *i* ^2^ = 3.91, *p* = 0.27), suggesting quite consistent results across studies. This consistency, despite the fact that one cannot entirely exclude variability in the definition of “other infections” and variability in perioperative protocols across studies, makes the pooled estimate more trustworthy.

**Figure 7:**
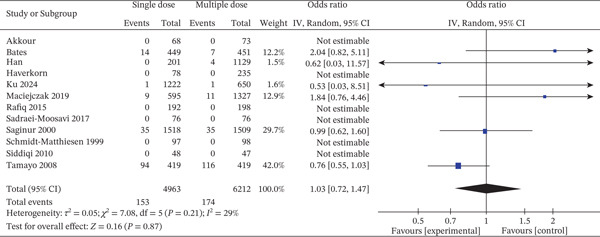
Forest plot of mortality incidence comparing single‐dose versus multiple‐dose prophylactic antibiotics (12 studies; random‐effects model). The pooled effect estimate is presented as ORs with 95% CIs. Statistical heterogeneity was assessed using the I^2^ statistic. The *x*‐axis represents ORs on a logarithmic scale.

This meta‐analysis combined data from 12 studies that published mortality outcomes in patients who had received single‐dose or multiple‐dose perioperative antibiotic prophylaxis (Figure 7). There were 153 deaths among 4963 patients in the single‐dose group and 174 deaths among 6212 patients in the multiple‐dose group. The summary OR for mortality was 1.03 [95% CI: 0.72–1.47], corresponding to no statistically significant mortality difference between the two regimens (*Z* = 0.16, *p* = 0.87). The CI traversed the null value (*OR* = 1), which also indicated the absence of apparent mortality benefit for single and multiple doses.

Among the studies with estimable ORs, Bates et al. (OR: 2.04 [0.82–5.11]) found a non‐significant trend for higher mortality in the single‐dose group, while Tamayo et al. (OR: 0.76 [0.55–1.03]) found a potential but not statistically significant protective effect of single‐dose prophylaxis [14, 50]. The largest and most powerful study, Saginur et al., contributed 29.7% of the weight and found an OR of 0.99 [0.62–1.60], which further supported the overall null effect [43]. Several trials, including Han et al., Ku et al., Maciejczak et al., and Schmidt‐Matthiesen et al., contained few or no events in one or both arms, so CIs were wide or effect sizes were not estimable. These trials contributed little to the analysis due to low event rates and sample sizes [23, 32, 35, 45]. Notably, Tamayo et al. contributed the most to the pooled estimate (42.0% weight) as it had a larger sample size and number of events [50]. Heterogeneity was low‐to‐moderate (*I* ^2^ = 29*%*, *T* *a* *u* ^2^ = 0.05, *C* *h* *i* ^2^ = 7.08, *p* = 0.21), indicating a moderate level of consistency across studies. Differences in study design, surgical populations, and definitions of mortality (e.g., 30‐day vs. in‐hospital) may have been responsible for the observed statistical dispersion.

**Figure 8:**
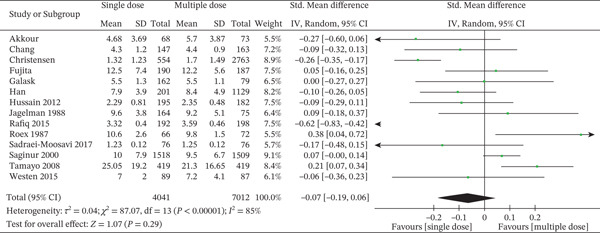
Forest plot of readmission rate incidence comparing single‐dose versus multiple‐dose prophylactic antibiotics (14 studies; random‐effects model). The pooled effect estimate is presented as ORs with 95% confidence intervals. Statistical heterogeneity was assessed using the I^2^ statistic. The *x*‐axis represents ORs on a logarithmic scale.

Fourteen studies compared single‐dose versus multiple‐dose antibiotic prophylaxis regimens in assessing hospital readmission rates (Figure 8). There were 153 readmission events among 4963 patients in the single‐dose arm and 174 events among 6212 patients in the multiple‐dose arm. Pooled OR for readmission was 1.03 [95% CI: 0.72–1.47], and it was not statistically significantly different between the two dosing regimens regarding risk of readmission (*Z* = 0.16, *p* = 0.87). The CI did not cross the no‐effect line, confirming that neither dosing approach provided a detectable benefit in averting readmissions.

The individual most strongly weighted contributor to the analysis was Tamayo et al., with a weight of 42.0% due to its very high event rate and sample size. The OR from this study was 0.76 [0.55–1.03], suggesting there could be fewer readmissions with the single‐dose approach, although this did not reach statistical significance [50]. Bates et al. had a different OR of 2.04 [0.82–5.11], this time in favor of multiple doses, but again the CI encompassed the null value [14].

Some of the studies, for example, Han et al., Ku et al., and Maciejczak et al., were of little significance (< 2%) since they were small and had few events and therefore created wide and non‐significant CIs [23, 32, 35]. Notably, Saginur et al., another high‐weight study (29.7%), presented an OR of 0.99 [0.62–1.60], which again reinforced the pooled null effect [43]. Heterogeneity was moderate (*I* ^2^ = 29*%*, *T* *a* *u* ^2^ = 0.05, *C* *h* *i* ^2^ = 7.08, *d* *f* = 5, *p* = 0.21), suggesting some heterogeneity of study populations or readmission definitions but not large enough to threaten the general consistency of evidence.

## 4. Discussion

This systematic review and meta‐analysis contrasted the relative effectiveness of single‐dose versus multiple‐dose prophylactic antibiotic regimens in a wide range of surgical specialties. Based on 47 trials involving thousands of patients, this review aimed to determine whether extending prophylactic antibiotics beyond a single preoperative dose offers additional benefit in reducing SSIs, postoperative morbidity, or mortality. Across a wide range of surgical specialties, the pooled evidence suggests no statistically significant reduction in SSIs with multiple‐dose prophylactic antibiotic regimens compared with single‐dose administration. These findings are broadly consistent with current international guidelines, which recommend single‐dose prophylaxis for most clean and clean‐contaminated procedures. However, the observed absence of statistical significance should be interpreted cautiously and does not imply definitive clinical equivalence across all surgical contexts.

Several well‐powered trials replicated this finding. For example, Bates et al. in their report of a RCT of implant‐based breast reconstruction found no difference between patients on single‐dose cloxacillin and those on an extended course of infection rate [14]. Similarly, Gahm et al. from a large group of pediatric neurosurgical patients found no obvious advantage of multiple‐dose cephalothin over a single dose, with both groups having low infection rates [19]. These findings agree with the principle of minimum effective exposure, the cornerstone of antimicrobial stewardship. Others such as Abro et al. and Sadraei‐Moosa et al., however, reported moderate rates of reduction in infection rates with prolonged dosing in high risk categories, for example, CABG or colonic surgery, where risk of contamination is assumed to be higher [9, 42]. Sadraei‐Moosavi et al., for example, noted that multiple‐dose cefmetazole had a marginally lower incidence of infections compared with single preoperative dose regimens despite the absolute difference being very small and nonsignificant [42]. These occasional messages suggest that while single‐dose regimens are sufficient for most procedures, individualization of prophylaxis to the surgical site and patient risk pattern remains clinically prudent.

Mortality rates were low across the majority of studies and did not differ meaningfully between dosing regimens. For instance, Tamayo et al. reported 30‐day and 90‐day mortality rates of 5.2% and 6.9%, respectively, with no evident benefit to extended prophylaxis [50]. In studies such as Ku et al. and Gahm et al., mortality was minimal or even not recorded, likewise suggesting that extended use of antibiotics has a minimal, if any, impact on survival rates among elective surgical patients [19, 32].

Interpretation of these findings must account for the substantial clinical and methodological heterogeneity observed across included studies. Variability was evident in surgical type, degree of wound contamination, antibiotic class and dosing schedules, perioperative protocols, follow‐up duration, and definitions of SSI. Such heterogeneity likely contributed to the moderate I^2^ value observed in the primary analysis and limits the extent to which pooled estimates can be applied to specific surgical subgroups.

Readmission and reoperation rates, though occasionally reported, were similarly not found to have a systematic benefit in either direction for either dosing strategy. For example, Christensen et al. and Bashir et al. each had comparable rates of post‐discharge infection and readmission across groups, pointing out that prolonged prophylaxis did not provide quantifiable downstream benefit [13, 16]. Also, Haider et al. and Saginur et al. demonstrated that with the implementation of standard surgical technique and perioperative care, prevention of infection was possible without multi‐dose regimens [22, 43].

The choice of antibiotic was different in the studies, but first‐generation cephalosporins, particularly cefazolin, were the most commonly used agents. This is in accordance with guideline recommendations due to their narrow spectrum, favorable safety profile, and adequate tissue penetration. Other studies substituted alternatives like vancomycin, ciprofloxacin, or metronidazole in the allergic subject or in some surgical interventions (e.g., neurosurgical or gastrointestinal interventions). Interestingly, some of these studies using broad‐spectrum or combination regimens also were able to find no benefit of multiple dosing, stating that antibiotic choice should be sufficient but not in excess [23, 55]. The heterogeneity of studies by surgical category, antibiotic regimen, and outcome definition was impressive, but uniformity of overall results lends strength to the generalizability of the results. Even so, it must be noted that some studies had less than complete reporting of side effects, microbiological confirmation of infection, or long‐term follow‐up, potentially limiting full assessment of the clinical meaning of prolonged use of antibiotics.

Several well‐powered trials reported comparable infection rates between single‐dose and extended prophylactic regimens. While these findings are consistent with the principle of minimum effective antibiotic exposure, some studies conducted in higher‐risk surgical settings reported trends favoring prolonged prophylaxis. Importantly, these differences were generally small and not statistically significant, underscoring the need for cautious interpretation rather than definitive conclusions. From an antimicrobial stewardship perspective, these findings support existing recommendations advocating for the shortest effective duration of prophylactic antibiotic exposure. Unnecessary extension of prophylaxis may increase the risk of adverse drug effects and antimicrobial resistance without demonstrable clinical benefit. This consideration is particularly relevant in low‐ and middle‐income settings, where prolonged antibiotic use remains common and stewardship resources may be limited.

Cumulatively, these findings further support the increasing international emphasis on antimicrobial stewardship. Widespread use of multiple‐dose regimens for which benefit is not demonstrable not only exposes patients to unnecessary risk of antibiotic‐related diarrhea, resistance, and reaction, but also contributes to the growing global threat of antimicrobial resistance. In concluding that single‐dose prophylaxis is sufficient to prevent infection in most surgical cases, this review provides compelling evidence to justify rational prescribing and judicious use of antibiotics.

### 4.1. Limitations of Included Studies

There were a number of limitations identified throughout the included studies. Firstly, heterogeneity regarding study design, antibiotic regimens, and outcome definitions made direct comparisons unfeasible. Some of the studies lacked standardized SSI definitions or secondary outcomes such as reoperations or microbiologic confirmation. Secondly, follow‐up duration and timing were extremely variable, with the majority of studies limiting follow‐up at 7–30 days, potentially leading to underestimation of late‐onset infection. Thirdly, antibiotic classes and doses were variable and allergen substitutions were not always clearly reported. In addition, blinding and concealment of allocation were not universally applied in randomized trials, and few observational studies did not control for confounders, the introduction of bias.

The classification of ‘multiple‐dose’ prophylaxis encompasses a wide spectrum of clinical practices, from limited postoperative dosing to extended multi‐day regimens. Because of inconsistent and incomplete reporting of dosing duration across studies, it was not possible to stratify analyses according to prophylaxis length. This limitation reduces the granularity of the comparison and should be considered when translating findings into clinical practice.

The substantial statistical heterogeneity observed in the primary analysis (*I* ^2^ = 65*%*) reflects profound underlying clinical heterogeneity across included studies. Procedures ranged from clean, elective operations with inherently low baseline infection risk to complex, high‐risk surgeries involving contaminated fields and emergency settings. As such, the pooled effect estimate should not be interpreted as evidence of clinical equivalence between single‐dose and multiple‐dose prophylaxis across all surgical contexts. Rather, it represents an average effect across diverse settings, and important procedure‐specific differences may exist that cannot be reliably isolated using the available data.

### 4.2. Clinical Takeaway for Clinicians

The available evidence suggests that a single preoperative dose of prophylactic antibiotics is sufficient for SSI prevention in many operative settings. Across diverse surgical disciplines including orthopedic, gynecologic, gastrointestinal, and cardiovascular procedures pooled analyses did not demonstrate a statistically significant advantage of extended prophylactic regimens when results were averaged across heterogeneous populations. However, this finding should not be interpreted as evidence of clinical equivalence in all surgical contexts.

Prolonged antibiotic prophylaxis should therefore be reconsidered in light of its uncertain incremental benefit and its potential to increase adverse effects, costs, and antimicrobial resistance. Individualized decision‐making remains essential, particularly in patients with elevated infection risk (e.g., immunosuppression, contaminated or emergency procedures, implant‐based surgery), where extended dosing may still be clinically justified. Overall, these findings support a stewardship‐oriented approach that favors the shortest effective duration of prophylaxis while accounting for patient‐ and procedure‐specific factors.

### 4.3. Future Directions and Implications

Future studies should be directed towards large, multicenter RCTs that stratify patients by surgical type, degree of contamination, and host risk factors in order to determine when long‐term antibiotic prophylaxis is indicated. Studies should utilize homogeneous definitions of SSI and uniform follow‐up intervals to maximize comparability. Future studies should also measure harms due to antibiotics, including resistance patterns, adverse drug effects, and alterations of the microbiome. Cost‐effectiveness analyses of single‐ vs. multiple‐dose regimens in different low‐ and middle‐income country health systems will be paramount for global acceptance of optimized prophylactic approaches.

## 5. Conclusion

This systematic review and meta‐analysis demonstrates that single‐dose antibiotic prophylaxis is as effective as multi‐dose regimens in preventing SSIs across a wide spectrum of surgeries. Extended antibiotic use did not improve infection‐related outcomes and may contribute to unnecessary costs, adverse effects, and antimicrobial resistance. These findings strongly support guideline‐concordant single‐dose protocols in most routine surgeries. While certain high‐risk patients or procedures may warrant individualized consideration, prolonged prophylaxis should not be the default. Adoption of single‐dose strategies can enhance patient safety, reduce antibiotic overuse, and align clinical practice with global stewardship efforts.

## Author Contributions

All authors made significant contributions to this research in the form of study design, acquisition of information, drafting, revising and critically reviewing the manuscript.

## Funding

No funding was received for this manuscript.

## Disclosure

All authors approve the publication of the final version of the manuscript.

## Ethics Statement

The authors have nothing to report.

## Conflicts of Interest

The authors declare no conflicts of interest.

## Supporting information


**Supporting Information** Additional supporting information can be found online in the Supporting Information section. The Supporting Information contains the search strategy that was used to obtain the results for this review. It also contains the risk of bias assessment tables for the reviewers to have a clear idea of how the assessment was carried out.

## Data Availability

Data sharing is not applicable to this article as no new data were created or analyzed in this study.
