# Supplementary material for: Comparative Effectiveness of Single‐Dose vs. Multi‐Dose Prophylactic Antibiotics in Reducing Post‐Surgical Infections: A Systematic Review and Meta‐Analysis
Source: Int J Microbiol. 2026 May 21;2026:8368761. doi: 10.1155/ijm/8368761 (PMC13191826; doi:10.1155/ijm/8368761)
Supplement: Supplementary file 1 — Supporting Information Additional supporting information can be found online in the Supporting Information section. The Supporting Information contains the search strategy that was used to obtain the results for this review. It also contains the risk of bias assessment tables for the reviewers to have a clear idea of how the assessment was carried out. [file IJM-2026-8368761-s001.docx]

***Title: Comparative Effectiveness of Single-Dose vs. Multi-Dose Prophylactic Antibiotics in Reducing Post-Surgical Infections: A systematic review and meta-analysis***

***Sariya Khan^1^, Mariam Amro^1^, Muddassir Khalidi^2^, Sadia Sultana^1^, Mable Pereira^3^, Manal ElSaid^4,5^***

**PICO Framework**

**P-** Patients undergoing any type of surgery

**I-** Single dose of prophylactic antibiotic

**C**- Multi-dose of prophylactic antibiotic

**O**- Reduction in post-surgical infections

**Search Strategy:**

(("Prophylactic Antibiotics" OR "prophylactic antibiotic*" OR "antibiotic prophylaxis")

AND ("single-dose" OR "single dose" OR "single administration" OR "single-shot antibiotic")

AND ("multi-dose" OR "multiple-dose" OR "multiple doses" OR "repeated doses")

AND ("post-surgical infection*" OR "surgical site infection" OR "SSI" OR "postoperative infection*"))

**Risk of Bias Tables**

***Table S1. Review authors' judgments about each risk of bias item for each included study.***

|  | Bias arising from the randomization process | Bias due to deviations from intended interventions | Bias due to missing outcome data | Bias in measurement of the outcome | Bias in selection of the reported result | Overall RoB |
| --- | --- | --- | --- | --- | --- | --- |
| **Tamayo E et al. (2008)** | Some concerns | Low | Some concerns | Low | Low | Low |
| **Hellbusch LC et al. (2008)** | Some concerns | Low | Low | Low | Low | Low |
| **Gahm J et al. (2022)** | Low | Low | Low | Low | Low | Low |
| **Sadraei-Moosavi SM et al. (2017)** | Some | Some concerns | Low | Some | Low | Some |
| **Rafiq MS et al. (2015)** | Some | High | Low | High | Some | High |
| **Lyimo FM et al. (2013)** | Low | Low | Low | Low | Low | Low |
| **Abro S et al. (2014)** | Low | Low | Low | Low | Low | Low |
| **Ishibashi K et al. (2014)** | Low | Low | Low | Low | Low | Low |
| **Westen EH et al. (2015)** | Low | Low | Low | Low | Low | Low |
| **Kanellakopoulou K et al. (2009)** | Low | Low | Low | Low | Low | Low |
| **Suzuki T et al. (2011)** | Low | Low | Low | Low | Low | Low |
| **Haga Y et al. (2012)** | Low | Low | Low | Low | Low | Low |
| **Lyimo FM et al. (2012)** | Low | Low | Low | Low | Low | Low |
| **Hussain A et al. (2012)** | Low | Low | Low | Low | Low | Low |
| **Siddiqi A et al. (2010)** | Low | Low | Low | Low | Low | Low |
| **Fujita S et al. (2007)** | Low | Low | Low | Low | Low | Low |
| **Mohri Y et al. (2007)** | Low | Low | Low | Low | Low | Low |
| **Jabeen N et al. (2007)** | Low | Low | Low | Low | Low | Low |
| **Shah M et al. (1998)** | Low | Low | Low | Low | Low | Low |
| **Bates T et al. (1992)** | Low | Low | Low | Low | Low | Low |
| **Saginur R et al. (2000)** | Low | Low | Low | Low | Low | Low |
| **Schmidt-Matthiesen A et al. (1999)** | Low | Low | Low | Low | Low | Low |
| **Roex AJ et al. (1987)** | Low | Low | Low | Low | Low | Low |
| **Jagelman DG et al. (1988)** | Low | Low | Low | Low | Low | Low |
| **Galask RP et al. (1988)** | Low | Low | Low | Low | Low | Low |

***The Methodological Index for Non-Randomized Studies (MINORS)***

Table S2. MINORS assessment tool for non-randomized non-comparative studies (n =2)

| **Item** | **Badge,2022^14^** | **Warnock,2019^19^** |
| --- | --- | --- |
| **A clearly stated aim** | 2 | 2 |
| **Inclusion of consecutive patients** | 2 | 2 |
| **Prospective collection of data** | 2 | 2 |
| **Endpoints appropriate to the aim of the study** | 2 | 2 |
| **Unbiased assessment of the study endpoint** | 1 | 1 |
| **Follow-up period appropriate to the aim of the study** | 1 | 1 |
| **Loss to follow-up less than 5%** | 1 | 1 |
| **Prospective calculation of the study size** | 0 | 0 |
| ***Total Score*** | 11 | 11 |

Table S3. MINORS assessment tool for non-randomized comparative studies (n = 20)

| **Item** | **Thareja, 2023 ^3^** | **Mangan, 2025^4^** | **Ku, 2024 ^5^** | **Bashir, 2024^7^** | **Zafar, 2024^8^** | **Akkour, 2020^9^** | **Fryberger, 2021^10^** | **Mohammadi, 2022^11^** | **Haider, 2022^12^** | **Christensen,2021^13^** | **Salih,2018^15^** | **Wyles,2019^17^** | **Maciejczak,2019^18^** | **Han,2014^20^** | **Andy,2014^25^** | **Chang,2008^30^** | **Kasatpibal,2006^35^** | **Sevin,2007^37^** | **Mehta,1990^47^** | **Haverkon,1987^43^** |
| --- | --- | --- | --- | --- | --- | --- | --- | --- | --- | --- | --- | --- | --- | --- | --- | --- | --- | --- | --- | --- |
| **A clearly stated aim** | 2 | 2 | 2 | 2 | 2 | 2 | 2 | 2 | 2 | 2 | 2 | 2 | 2 | 2 | 2 | 2 | 2 | 2 | 2 | 1 |
| **Inclusion of consecutive patients** | 1 | 1 | 1 | 1 | 1 | 1 | 1 | 1 | 1 | 2 | 2 | 1 | 2 | 2 | 1 | 1 | 1 | 1 | 1 | 1 |
| **Prospective collection of data** | 2 | 0 | 1 | 0 | 2 | 0 | 0 | 0 | 2 | 0 | 2 | 0 | 2 | 0 | 0 | 0 | 2 | 2 | 2 | 1 |
| **Endpoints appropriate to the aim of the study** | 2 | 2 | 2 | 2 | 2 | 2 | 2 | 2 | 2 | 2 | 2 | 2 | 2 | 2 | 2 | 2 | 1 | 2 | 2 | 2 |
| **Unbiased assessment of the study endpoint** | 1 | 1 | 1 | 1 | 1 | 1 | 1 | 0 | 1 | 1 | 1 | 1 | 1 | 1 | 1 | 1 | 1 | 1 | 1 | 1 |
| **Follow-up period appropriate to the aim of the study** | 2 | 1 | 2 | 1 | 1 | 1 | 1 | 1 | 1 | 2 | 1 | 1 | 1 | 1 | 1 | 1 | 1 | 1 | 1 | 1 |
| **Loss to follow-up less than 5%** | 2 | 1 | 2 | 2 | 0 | 1 | 0 | 1 | 1 | 2 | 1 | 2 | 1 | 0 | 0 | 2 | 0 | 1 | 0 | 0 |
| **Prospective calculation of the study size** | 0 | 0 | 0 | 0 | 0 | 0 | 0 | 1 | 0 | 1 | 0 | 0 | 0 | 0 | 0 | 0 | 1 | 0 | 0 | 0 |
| **An adequate control group** | 1 | 1 | 2 | 1 | 2 | 2 | 2 | 1 | 2 | 2 | 2 | 2 | 2 | 2 | 2 | 2 | 2 | 1 | 1 | 2 |
| **Contemporary groups** | 2 | 2 | 2 | 2 | 2 | 2 | 2 | 0 | 2 | 2 | 2 | 2 | 2 | 1 | 2 | 2 | 1 | 2 | 0 | 1 |
| **Baseline equivalence of groups** | 1 | 1 | 2 | 1 | 1 | 2 | 1 | 1 | 2 | 2 | 2 | 1 | 1 | 1 | 1 | 2 | 1 | 1 | 1 | 1 |
| **Baseline equivalence of groups** | 2 | 2 | 2 | 1 | 1 | 2 | 1 | 2 | 2 | 2 | 1 | 1 | 2 | 2 | 2 | 2 | 0 | 1 | 1 | 1 |
| ***Total Score*** | 18 | 14 | 19 | 14 | 15 | 16 | 13 | 12 | 18 | 20 | 18 | 15 | 18 | 14 | 14 | 17 | 13 | 15 | 12 | 12 |
